# Quantitative image analysis for evaluating the abrasion resistance of nanoporous silica films on glass

**DOI:** 10.1038/srep17708

**Published:** 2015-12-10

**Authors:** Karsten H. Nielsen, Stefan Karlsson, Rene Limbach, Lothar Wondraczek

**Affiliations:** 1 Otto Schott Institute of Materials Research, University of Jena, Fraunhoferstrasse 6, D-07743 Jena, Germany; 2 Glafo–the Glass Research Institute, PG Vejdes väg 15, SE-351 96 Växjö, Sweden; 3 Center of Energy and Environmental Chemistry (CEEC), University of Jena, Max-Wien-Platz 1, D-07743 Jena, Germany

## Abstract

The abrasion resistance of coated glass surfaces is an important parameter for judging lifetime performance, but practical testing procedures remain overly simplistic and do often not allow for direct conclusions on real-world degradation. Here, we combine quantitative two-dimensional image analysis and mechanical abrasion into a facile tool for probing the abrasion resistance of anti-reflective (AR) coatings. We determine variations in the average coated area, during and after controlled abrasion. Through comparison with other experimental techniques, we show that this method provides a practical, rapid and versatile tool for the evaluation of the abrasion resistance of sol-gel-derived thin films on glass. The method yields informative data, which correlates with measurements of diffuse reflectance and is further supported by qualitative investigations through scanning electron microscopy. In particular, the method directly addresses degradation of coating performance, i.e., the gradual areal loss of antireflective functionality. As an exemplary subject, we studied the abrasion resistance of state-of-the-art nanoporous SiO_2_ thin films which were derived from 5–6 wt% aqueous solutions of potassium silicates, or from colloidal suspensions of SiO_2_ nanoparticles. It is shown how abrasion resistance is governed by coating density and film adhesion, defining the trade-off between optimal AR performance and acceptable mechanical performance.

Thin films are omnipresent on flat glass substrates for applications in almost any area of daily life. Most prominently, they are used to improve or impose specific functionality on glass sheet in architecture, automotive engineering and solar energy harvesting, for example, to generate wavelength-specific reflectivity or anti-reflectivity (AR), electrical conductivity, photocatalytic activity and/or self-cleaning ability^1^. To increase light transmission in photovoltaic modules, the cover glasses are often equipped with nanoporous SiO_2_ AR coatings, which can be synthesized by vapor deposition processes^2^ or by sol-gel routes from potassium silicates^3^, silica nanoparticles^4^ or silicon alkoxides, such as tetraethyl orthosilicate (TEOS)^5^,^6^. For photovoltaic cover glasses in outdoor applications which are designed for a lifetime of up to 40 years^7^, chemical and mechanical stability are crucial arguments and have therefore been addressed continuously, with strategies ranging from increasing the precursor reactivity^5^, controlling the gel morphology^6^,^8^, partial crystallization^9^, enhancement of interfacial reactions for improving film adhesion^10^ to post-deposition treatments with gases or solutions^4^,^10^,^11^,^12^. Accurately judging and quantifying the mechanical stability (usually expressed as the abrasion resistance) of such coatings, however, remains a standing issue. In particular, today’s typical evaluation procedures, which either rely on instrumented indentation or on more simplistic but standardized macroscopic tests, do not provide direct insights into the degradation of actual performance (which is, for AR coatings, the ability to improve optical transparency over a certain area of glass sheet). The former approaches for evaluating the mechanical properties of thin films on glass involve advanced and precise but very local testing through nanoscratching or nanoindentation^13^,^14^,^15^,^16^,^17^. The latter, macroscopic techniques range from wipe tests^12^,^18^,^19^, pencil hardness tests^9^,^20^ or adhesive tape tests^10^,^21^ to controlled abrasion such as done with the widely-employed rotating abrader (Taber® Abraser), or with linear abraders^5^. A major objective of these tools is to mimic, in one way or the other, real-world contact damage. Laboratory abrasion is then typically followed by optical inspection, which usually addresses local defects and aims to determine a critical threshold load or parameter, which causes visible scratching of the coating^9^ or coating detachment^22^. A rotating abrader is schematically shown in Fig. 1. It comprises rotating abrasive wheels which are equipped with a selectable surface material for mimicking specific contact situations. Further adjustable parameters are the contact load, the rotation speed, and the number of abrasion cycles. This setup complies with several standards for transparent materials and glazed materials^23^,^24^. For example, it is applied regularly for empirically testing of coated glass sheet and the mechanical resistance of sol-gel^5^,^25^,^26^ as well as physical vapor deposition (PVD)-derived coatings^27^. The abrasive wheels typically consist of either felt^5^ or a polymer matrix with embedded corundum (Al_2_O_3_)^24^. After abrasion, the response of the coated surface is often judged through mass loss^26^, or by recording optical parameters such as the direct or diffuse transmission^8^,^27^, the solar-weighted photon spectrum^5^ or haze^25^. The mechanical test is then often followed by microscopic investigations for a qualitative evaluation of the underlying abrasion process^22^. However, any such approach remains largely phenomenological, and the potential for drawing quantitative conclusions is limited. In particular, the comparison of different materials and different abrasion situations is complicated by the present inability to directly provide a facile numerical evaluation of areal surface degradation.

In the present study, we target this issue through combining quantitative image analysis with standard abrasion tests. We show that using mature tools of computational image analysis on scanned micrographs enables rapid determination of areal variations in the optical properties of the coated substrate area after abrasion. The sensitivity of this approach is limited only by the quality of the image collection, enabling to resolve even small variations that occur between comparably similar coatings, or between small steps of mild abrasion. For this, we consider two kinds of SiO_2_ sol-gel coatings as model systems: films based on colloidal SiO_2_ with adjustable colloid size and comparably weak adhesion^28^, and more durable films which are derived from the deposition of aqueous potassium silicate solutions^3^,^29^ with varying film density.

## Materials and Methods

### Sample synthesis

Nanoparticle-derived layers were produced from commercial, alkaline suspensions of colloidal SiO_2_ (Köstrosol®, CWK Bad Köstritz, Bad Köstritz, Germany). Different suspensions with particle diameters of 7, 20, 35 and 45 nm, respectively, were used. The suspensions were adjusted to pH = 7 with 0.1 M HCl, subsequently diluted to a solid fraction of 6 wt%, and immediately applied to 2 mm low-iron float glass (*T*_g_ = 566 °C) by dip-coating (RDC 15, Bungard Elektronik, Windeck, Germany) to generate homogeneous coatings such as previously described by Cook^28^. For the solution-derived films, aqueous potassium silicates (BASF, Düsseldorf, Germany) with a nominal silicate concentration of ~6 wt% were employed (for details, see Refs. 3,29). Here as well, coatings were deposited by dipping under ambient atmosphere, followed by subsequent washing in demineralized water so as to remove residual alkaline carbonates^3^. After drying, both types of samples were thermally annealed for 20 minutes at 500 °C on graphite plates in a muffle furnace (LM 312, Linn High Therm GmbH, Eschenfelden, Germany) with the tin-side of the glass substrate facing upwards. Both types of precursor yield thin SiO_2_ films with AR functionality. Film thickness *d* and refractive index *n*_p_ of the coatings were estimated through ellipsometric analysis (EP3, Accurion GmbH, Göttingen Germany). For that, measurements were performed at several angles and a fixed wavelength of *λ* = 514 nm. Data on these coatings are summarized in Table 1.

### Microindentation

An empirical indication of the mechanical stability of thin films can be derived from the probability of radial crack formation after Vickers indentation, the so-called crack propensity index (*CPI*)^30^,^31^. This comprises two separate effects: (i) the effect of reduced contact-stress during indentation through a coating which is soft, when compared with the substrate, and (ii) the effect of tensile (or compressive) Eigen-stress in the coating as generated through strain differences which occur relative to the substrate upon coating consolidation. Both effects lead to variations in *CPI*, which are caused by the coating but occur in the substrate. Vickers indents (Duramin-1, Struers, Ballerup, Denmark) were made on the coated tin-side of the glasses, and the number of radial cracks formed at the corners of the residual Vickers hardness imprints was determined by optical microscopy 20 seconds after indentation. The measurements were conducted under ambient atmosphere at 5 different loads between 50 and 500 g and were repeated 20 times for each load.

### Abrasion analysis

The coated glass samples were prepared for abrasion testing by washing under tap and distilled water and subsequent drying with pressurized air. The prepared samples were kept upright in closed plastic boxes before the abrasion experiment in order to ensure uniform conditioning of the surface and to protect them from dust. All abrasion tests were conducted on the coated tin-side of the glass to minimize the influence of any eventual glass substrate corrosion^1^.

**Figure 1:**
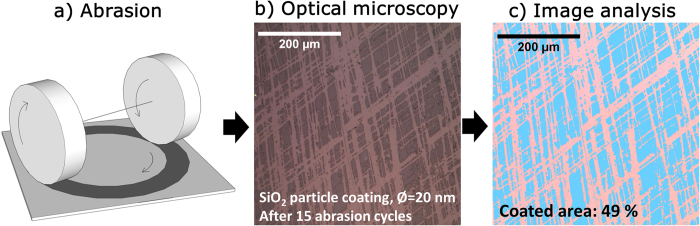
Principle of the test method. Thin film coated glass is abraded with the Taber® abraser (**a**) above the critical load to cause delamination. An optical microscope is used to collect images from the middle of the abraded surface area (**b**). For anti-reflective coatings, the coating and substrate can easily be distinguished. Finally image analysis (**c**) is used to determine the average coated area. This procedure is repeated several times for each sample.

Abrasion experiments were performed in a rotating-wheel abrader (Fig. 1a, Abraser 5135, Taber Industries, North Tonawanda, USA). In the following, one complete 360° rotation of the sample is called an abrasion cycle. Initially, a rotation speed of 60 rmp under no additional load with Cs-10f abrasive wheels was chosen as reference condition. These abrasion wheels consisted of Al_2_O_3_ particles in a polymer matrix^24^, and represent a typical benchmark for thin-film testing^23^,^26^,^27^. All particle-derived coatings where abraded with this set-up. Following initial reference testing, more violent abrasion conditions were employed for further testing of the potassium silicate-derived coatings, i.e., using a coarser Cs-10 abrasive wheel at 72 rpm and with an additional load of 750 g on each wheel. After a certain number of cumulative abrasion cycles, samples were demounted, rinsed with ethanol and drying again with pressurized air. Digital images of the thus-abraded samples were taken with an optical microscope (Axiolab, Zeiss, Oberkochen, Germany, 20× lens CP-Achromat, Zeiss, Oberkochen, Germany) in reflectance mode, equipped with a simple camera (Microcam 3 M, Bresser, Borken, Germany). With this microscope, images were collected from three arbitrary positions on the abraded surface, while microscope settings were optimized for maximum contrast in each picture (shown exemplarily in Fig. 1b). After image recording, abrasion was continued as described above, repositioning the sample into the abrader. Each test was repeated 2–3 times for any type of sample. Between different samples, the abrasive wheels were refreshed according to standard prescriptions. Image analysis was conducted with the Axiovision software (Zeiss, Oberkochen, Germany) with the goal to determine the extent of areal abrasion as a function of the number of abrasion cycles (Fig. 1c). Thereby, it was assumed that image recording provides direct access to the areal increase in surface reflectivity (or loss in sample transmission) which follows local removal of the AR layer upon abrasion. As a prerequisite, abrasion treatment was done so as to not damage the underlying glass substrate, which would lead to an increase in diffuse reflection and scattering, and would compromise the degree of information which can be extracted from the data for performance of the coating alone.

### Reference testing

For reference, further experiments were conducted to judge the abrasion resistance by conventional means^17^, and to verify the sensitivity and selectivity of image analyses. These experiments involved collecting diffuse reflection (*DR*) spectroscopy (using a Cary 5000 spectrophotometer, Agilent, Santa Clara, US, equipped with a 110 mm integration sphere and a goniometer for angle-sensitive analyses) and instrumented indentation testing, using a Nanoindenter G200 (Agilent, Santa Clara, US, equipped with a Berkovich diamond tip with a nominal tip radius of 50 nm). In order to increase the instrumental resolution at very shallow indentation depths the indenter tip was calibrated based on its equivalent contact radius^32^. Depth profiles of the hardness *H* and elastic modulus *E* were obtained by operating in the continuous stiffness measurement mode (CSM)^33^. Values of *H* and *E* were extracted at an indentation depth of ~40 nm, where substrate-independent values of *E* were achieved through the method proposed by Hay and Crawford^34^.

Furthermore, the abrasion wear resistance was investigated using the same nanoindenter as described above (but equipped with a conical diamond tip with a nominal tip radius of 5 μm). In this test the indenter tip was scratched multiple times across the sample surface along a distance of 200 μm at a constant load of 10 mN and a velocity of 50 μm/s. After each five consecutive wear cycles the topography of the residual scratch groove was scanned with the indenter tip at a constant load of 50 μN along as well as perpendicular to the wear path.

Finally, a scanning electron microscope (SEM) was used to investigate selected samples after abrasion (JSF7001F, Jeol Ltd., Tokyo, Japan). For this, samples were coated with a thin carbon layer prior to micrograph collection (Auto 306, Edwards, Crawley, United Kingdom).

## Results and Discussion

### Coating microstructure

The microstructure of the employed coatings is taken as the predominant factor for leveraging a broad variability of abrasion resistance. This is to judge the applicability and selectivity of digital image analyses in the quantification of areal abrasion damage. The most versatile design parameters are the coating porosity (which directly determines its effective refractive index) and interfacial adhesion. Then, the two employed types of coatings represent the extremes of low porosity and strong interfacial bonding (solution-derived films) and high porosity/weak bonding (films which are derived from colloidal suspensions). In the latter, interfacial adhesion is primarily governed by the degree of thermal curing and sintering, which again results in lower porosity. Beyond these extreme variations, fine variations are obtained within the individual classes of coatings through adjusting either the solution concentration and, in particular, the ratio of SiO_2_/K_2_O, or the colloid size.

As summarized in Table 1, the applied synthesis procedures yield films with thickness ranging from 70 to 120 nm, for both kinds of precursors. The potassium silicate-derived films exhibit higher refractive indices (*n*_P_ = 1.43–1.46) than the films derived from SiO_2_ nanoparticles (*n*_P_ = 1.34–1.38), what indicates lower film density and, eventually, lower abrasion resistance. The porosity of the obtained films was estimated from equation(1):^35^


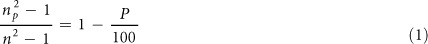


where *n* is the refractive index of the constituting material (for SiO_2_, *n* = 1.46), *n*_p_ the effective refractive index of the porous layer, and *P* the volume fraction of pores (%). The calculated porosity of the potassium silicate-derived thin films is comparably low, i.e., ~0–8%, while that of the SiO_2_ nanoparticle-derived films is much higher, i.e., ~20–30%, within the geometrical optimum of a system of close-packed, monodisperse balls (26%). Noteworthy, the absolute porosity value may be influenced by capillary condensation of water which occurs under ambient conditions^29^. Then, the value of *n*_p_ is strictly not a convolution of silica and air, but of silica and water with *n*_H2O_ ~ 1.33. In this case, the actual pore fraction would be higher than the here-employed estimate.

**Figure 2:**
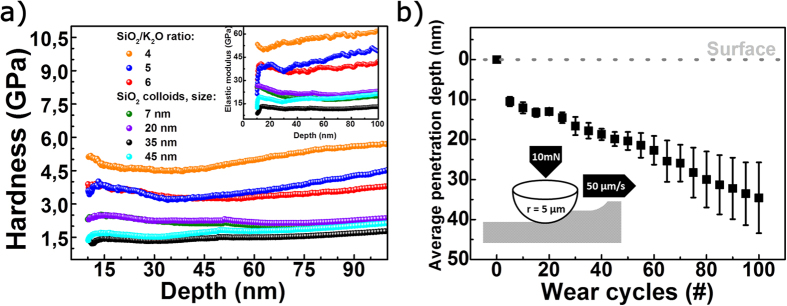
Nanoindentation and nano-wear experiments on the model coatings. (**a**) Hardness as a function of indentation depth for coatings based on potassium silicate solutions with varying SiO_2_/K_2_O molar ratios and solutions of SiO_2_ nanoparticles with different particle diameters, respectively. The inset shows the elastic modulus as a function of indentation depth. (**b**) Average penetration depth as a function of wear cycles for nano-wear experiments on a thin film derived from a potassium silicate solution with SiO_2_/K_2_O = 4. The inset illustrates the principle of the experiment.

The results of the nanoindentation testing, shown in Fig. 2a and summarized in Table 1, confirm the expectation, that the coatings based on potassium silicates in general have a higher hardness (*H* = 3.28–4.58 GPa) and elastic modulus (*E* = 36.2–55.6 GPa) than the more porous coatings based on SiO_2_ nanoparticles (*H* = 1.42–2.24 GPa; *E* = 12.0–21.3 GPa). The hardness values of the porous SiO_2_ nanoparticle coatings are in a range comparable to acid-catalysed TEOS-derived coatings with *P* = 35 and 37%, respectively, and tempered at 450 °C (*H* = 1.3 and 1.5 GPa)^17^. For the different types of coatings of this study, however, the nanoindentation measurements do not enable a clear ranking of the mechanical resistance, although the expected higher stability with lower SiO_2_/K_2_O ratio and lower particle size is indicated by the results. That is, these experiments do not readily distinguish the properties of the present coatings.

**Figure 3:**
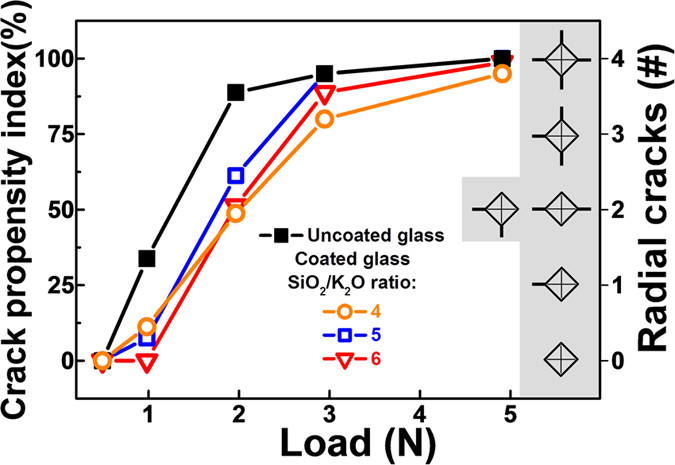
Crack propensity index for uncoated and coated glass. Crack propensity index as a function of indentation load for uncoated glass and potassium silicate coated glasses. Glasses are less prone to cracking upon coating. The inset illustrates radial crack formation upon Vickers indentation.

Using lateral-force control and/or measurement, instrumented nanoindentation enables an alternative method for testing the abrasion resistance of coatings, demonstrated exemplarily in Fig. 2b. With increasing number of wear cycles the indenter tip progressively penetrates deeper into the coating, if the applied load is above the critical load for the coating to fail. However, such wear experiments, are very time consuming, limited to relatively small observation length-scale and 1D-analyses, and still depend on the availability of highly accurate models for data evaluation. Figure 3 exemplarily shows the variation of *CPI* versus indentation load for the uncoated substrate and glass coated with potassium silicates of different SiO_2_/K_2_O molar ratios, that is SiO_2_/K_2_O = 4, 5 and 6, respectively. In general, for all coatings which are derived from potassium silicates, the crack resistance, in terms of the force which is needed to obtain a 50% probability (*CPI*_50_) for radial crack initiation in the substrate, is seen to increase from 1.3 N for the uncoated substrate to 1.8–2.0 N for the coated glasses, as summarized in Tab. 1. This is a significant improvement of surface defect resistance upon sharp contact loading, but does not necessarily let expect a notable variation also in abrasion resistance (blunt loading and lateral damage). The general phenomenon has been described earlier as a result of tensile stresses in the coatings, which, upon sufficiently strong film adhesion, act negatively on the crack-opening probability at the underlying substrate. Beyond the scope of the present study, this can be tailored through precursor dilution (coating density), film thickness, thermal treatment and the initial state of the glass surface^30^. Besides the generation of Eigen-stresses, also the sealing of defects has been noted as another factor in the improvement of surface damage resistance^14^,^21^,^36^. On the other hand, the data in Fig. 3 demonstrate that *CPI* does not provide sufficient selectivity to differentiate between the effects of the different types of coatings (with varying SiO_2_/K_2_O ratios), but only reveals a general reduction of surface defect sensitivity.

### Abrasion testing

**Figure 4:**
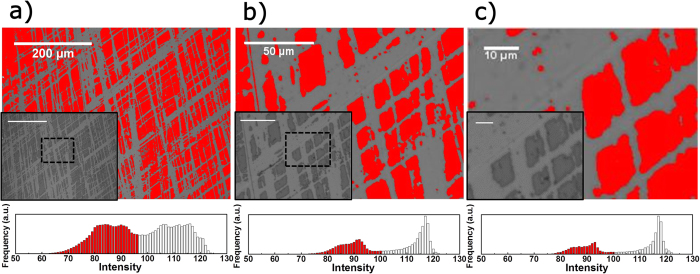
Image analysis of the abraded surface on different length scales. For each length scale, the segmented picture, grey scale original and the corresponding grey scale histogram are given as a basis for segmentation. (**b,c**) are obtained by cropping from (**a**), as marked on the insets. Sizes: (**a**) 588 × 441 μm (**b**) 163 × 122 μm (**c**) 65 × 49 μm.

As shown in Fig. 4a–c, picture analysis enables a clear distinction between the areas where the coating is removed (light) and the area with intact coating, which appears darker due to the AR properties of the coating. The observation length scale (Fig. 4b,c) largely depends on the applied imaging method and, in particular, its numerical aperture (NA). For rapid sample throughput and intermediate optical resolution, commercial flat-bed scanners can be employed with typically provide low NA. Shown here, however, are images taken with an optical microscope, as mentioned in the experimental section.

### Solution-derived coatings

**Figure 5:**
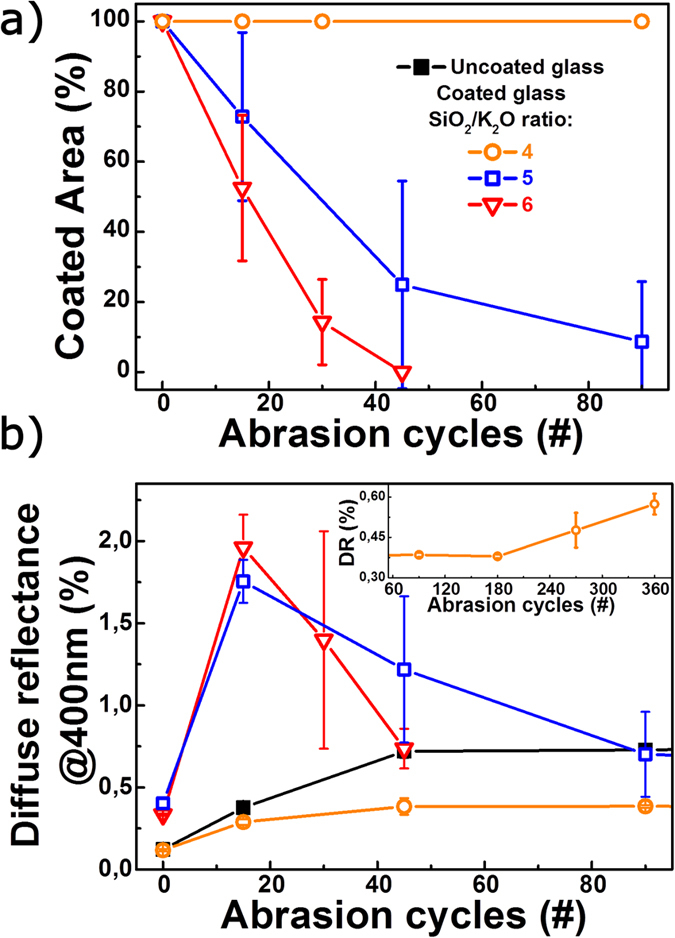
Abrasion testing of potassium silicate-derived films. (**a**) The average coated area as a function of abrasion cycles. (**b**) Diffuse reflectance (*DR*) evaluated at 400 nm as a function of number of abrasion cycles. The inset displays the development of *DR* for a coating with SiO_2_/K_2_O = 4 at higher numbers of abrasion cycles.

**Figure 6:**
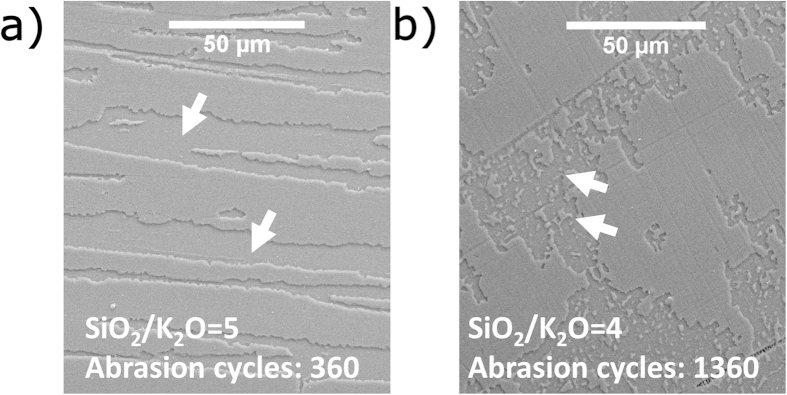
Scanning electron microscopy of abraded surfaces. The micrographs visualize the influence of the SiO_2_/K_2_O molar ratio on the wear behavior of potassium silicate coatings. (**a**) Thin film (SiO_2_/K_2_O = 5) after 360 abrasion cycles. The arrows exemplarily mark the regions where the coating is peeled off. (**b**) Thin film (SiO_2_/K_2_O = 4) after 1360 abrasion cycles. Remaining pieces of coating, marked by arrows, indicate higher interfacial strength.

The abrasion resistance of the solution-derived coatings was characterized by means of average coated area after a given number of abrasion cycles as shown in Fig. 5a. In contrast to the *CPI* analyses, image analyses enables a clear differentiation of the coatings with significantly increasing abrasion resistance for decreasing SiO_2_/K_2_O ratio. As summarized in Table 1, decreasing SiO_2_/K_2_O ratio leads to decreasing film porosity, probably due to the presence of more depolymerized precursor anions^3^. Interestingly, for coatings with SiO_2_/K_2_O ≥5, we observe removal from the surface through gradual delamination, whereas the coating with SiO_2_/K_2_O = 4 is sufficiently adherent to the surface so that the coating itself is abraded. Complementary microscope investigations confirm this observation (to be explained later, Fig. 6), where this most dense coating remains visually unaffected even after 90 cycles of rough abrasion, and notable scratches and signs of delamination are seen only after >200 cycles.

The corresponding evolution of diffuse reflectance (*DR*), at a wavelength of 400 nm, is shown in Fig. 5b. As noted previously, measurement of *DR* is a common tool for evaluating the progress of abrasion on coated or uncoated glass surfaces. Here, with progressing abrasion, all samples initially exhibit an increase in *DR*, which corresponds to the roughening of the surface. This effect is particularly pronounced for the samples with SiO_2_/K_2_O ≥ 5, where abrasion is primarily governed by coating delamination. However, after reaching a maximum in *DR* the *DR* starts to decrease and only when the coating is virtually completely removed, the value of the bare (uncoated) glass is reached. For the more dense coating with SiO_2_/K_2_O = 4 and for the bare glass, a similar but less pronounced increase in *DR* is seen, and a plateau is reached after prolonged abrasion. Hence, when *DR* is taken as a measure of abrasion-induced surface damage, the coating with SiO_2_/K_2_O = 4 apparently protects the surface against scratches. This is in contrast to the information, which is provided by *CPI* analyses, where all three solution-derived coatings were found to prevent the formation of radial cracks to approximately the same extent (Fig. 3), at least upon sharp contact loading. As intermediate conclusions, while analyses of *DR* may provide some information on the mechanism of abrasion (delamination versus gradual material removal), it is not an unambiguous way to judge the gradual and areal loss of AR functionality, in particular, as is obtained by digital image analysis.

Qualitative SEM analysis confirms this picture. Thin film failure occurs in two principle ways, i.e., due to failure of film adhesion or film cohesion^22^, exemplarily illustrated in Fig. 6a,b for solution-derived samples with SiO_2_/K_2_O = 5 and 4, respectively. The SEM micrograph in Fig. 6a was obtained from the edge of the former after 360 abrasion cycles, revealing film delamination without characteristic scratches on the intact parts of the remaining film fraction. The removal of the coating from the surface clearly point to adhesion failure, where the cracked or deformed edges indicate wedge spallation^22^. For comparison, the latter, more-dense coating (Fig. 6b) also starts to delaminate after extended abrasion (1360 cycles), with an increase in *DR* after 180 cycles (shown in the inset, Fig. 5b). However, pronounced individual scratches are also evident on the parts of coating which did not delaminate. These scratches are the reason for the comparably weak increase in *DR* in the early abrasion stages, as shown in Fig. 5b. The observations confirm that the latter film abrades through cohesive failure, and that adhesive failure comes into play only in the later stages of abrasion. However, also after prolonged abrasion, even very small islands of coating remain adherent to the surface (Fig. 6b, indicated with arrows).

### Colloid-derived coatings

**Figure 7:**
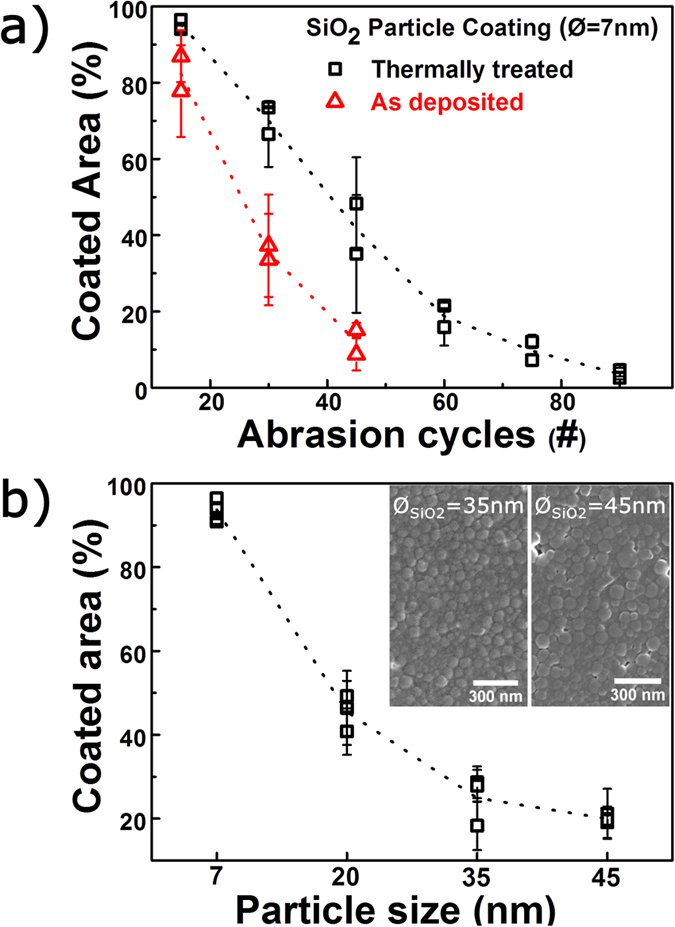
Investigations on SiO_2_ colloidal coatings. (**a**) The average coated area as a function of abrasion cycles for 7 nm SiO_2_ nanoparticle coatings. The line is a guide for the eye. (**b**) Coated area as a function of particle size for SiO_2_ nanoparticle coatings evaluated after 15 abrasion cycles. The line is a guide for the eye. The insets show SEM micrographs of two coatings, prepared from 35 nm and 45 nm particles, respectively. For both (**a,b**), two or more data points corresponds to two or more repeats of the same abrasion experiment.

As shown in the exemplary study of colloid-derived coatings, digital image analysis can also be employed to films with comparably low mechanical resistance, where other techniques, such as nanoindentation, fail to yield selective data (Fig. 2). Figure 7a clearly shows differentiation between as-deposited and thermally set layers, exemplarily for a coating, which was derived from a suspension of SiO_2_ spheres with a diameter of 7 nm. As expected, the thermal treatment increases the abrasion resistance of the coating, which agrees with previous observations on similar types of coatings^6^. In Fig. 7b, the average coated area after 15 abrasion cycles is shown as a function of the particle size, which was employed in the precursor suspensions, after thermal treatment. Here, the average coated area decrease, i.e., the abrasion resistance decreases, with increasing particle size, and a clear differentiation is possible between all samples. The type of precursor particle does not only influence abrasion resistance, but also coating morphology as illustrated in the insets in Fig. 4b. The SEM micrographs show coatings derived from SiO_2_ particles with diameters of 35 and 45 nm, respectively. A complete sintering of SiO_2_ nanoparticles is size-dependent under ~40 nm and is not expected for these coatings due to the short duration and relatively low applied temperatures^37^. However, the increased abrasion resistance upon thermal treatment (Fig. 4a) indicates that solidification takes place up to a certain extend. The observed increase in abrasion resistance with decreasing particle size may thus derive from size size-dependent reactivity^37^, but decreased roughness must also be considered, as suggested for PVD-derived thin films^13^.

Despite sintering and similar properties in terms of thickness and porosity, abrasion resistance for both kinds of thin films is greatly influenced by the employed starting solutions. Similarly, coatings derived from methyl trimethoxy silane^25^ were found to show a decrease in their abrasion resistance with increasing degree of polymerization and those findings correlate quite well with both types of thin films investigated in the present study. For TEOS-derived coatings it was noticed, that sols with polymeric chain structures give more stable coatings than particles, due to a better contact between gel-forming particles^6^. This explanation may also be valid for the two investigated thin film types in this study. For potassium silicate coatings, coating adhesion increases with increasing potassium content. This may correlate with potassium ions being enriched in the interface between coating and substrate^3^, where they could enhance an interfacial reaction by diffusion^38^ or by reducing the melting point of the SiO_2_ gel^10^ and thereby increase the adhesion of the coating to the glass.

In general, the two tested types of thin films are very different in terms of their mechanical stability, but due to the convolution of physical reactions which form the phenomenological behavior of abrasion resistance, this difference can often not readily be assessed, even by in-depth studies through, e.g., instrumented indentation. As shown above, simple image analyses might sometimes be much more suitable, where samples in each of the studied series could be clearly be distinguished in terms of their abrasion resistance.

## Conclusions

Controlled abrasion followed by image analysis for determination of the average coated area is suggested as a fast and practical tool for evaluating thin film abrasion resistance. This evaluation method provides informative and highly selective data which correlates with measurements of diffuse reflection and qualitative evaluation by scanning electron microscopy. The method was applied on two exemplary types of SiO_2_ coatings derived from two different sol-gel precursors, for which even slight differences in the coating chemistry and morphology could be traced successfully by the present high-throughput method. That is, abrasion resistance of the potassium silicate thin films was found to increase with decreasing SiO_2_/K_2_O molar ratio due to better coating adhesion, and that of SiO_2_ particle-derived coatings increases upon thermal treatment, and also with smaller particle size.

## Additional Information

**How to cite this article**: Nielsen, K. H. *et al*. Quantitative image analysis for evaluating the abrasion resistance of nanoporous silica films on glass. *Sci. Rep.* **5**, 17708; doi: 10.1038/srep17708 (2015).

## Figures and Tables

**Table 1:** Properties of the tested coatings on low iron float glass.

Potassium Silicate-Derived Thin Films				
*SiO*_*2*_*/K*_*2*_*O*	*4*	*5*	*6*	
*d* (nm)	110 ± 20	120 ± 20	110 ± 10	
*n*_p_	1.46 ± 0.02	1.46 ± 0.02	1.43 ± 0.01	
*P (%)*	0–5	0–5	5-10	
*T*_*increase*_*(%point)*	3.4	3.9	3.9	
*H*_*40 nm*_ *(GPa)*	4.58	3.26	3.28	
*E*_*40 nm*_ *(GPa)*	55.6	40.3	36.2	
***CPI_50_ (N)***	2.0	1.8	1.9	
SiO_2_ Nanoparticle-Derived Thin Films				
*Particle size (nm)*	*7*	*20*	*35*	*45*
*d* (nm)	93 ± 7	110 ± 12	70 ± 12	101 ± 5
*n*_p_	1.38 ± 0.02	1.36 ± 0.04	1.34 ± 0.04	1.36 ± 0.03
***P (%)***	20	25	30	25
***T_increase_ (%point)***	5.2 ± 0.3	6.5	6.5 ± 0.2	6.8
*H*_*40 nm*_ *(GPa)*	2.14	2.24	1.42	1.65
*E*_*4sm*_ *(GPa)*	18.5	21.3	12.0	17.7

Thickness (*d*), refractive index (*n_p_*), porosity (*P*) and maximum transmission increase (*T_increase_*) of a 2 mm low iron float glass (*T* = 91.4%). Hardness (*H*) and elastic modulus (*E*) as evaluated through nanoindentation. The force needed to obtain 50% crack propensity index (*CPI_50_*), this is 1.3 N for the uncoated glass substrate.
